# Eight Americas: A New Definition for “Americas”?

**DOI:** 10.1371/journal.pmed.0040042

**Published:** 2007-01-30

**Authors:** Howard Junca

The use of the term “Americas” in the context of this article [1] could confuse or mislead readers from a global audience. The authors used this term to refer to an artificial classification of United States (American) populations based on sociological aspects in order to group and analyse epidemiological information, but, Americas (plural and generally with the definite article) does not mean such division inside America (the United States of America in this case), but refers to the lands and regions of the Western hemisphere (North America, Central (or Middle) America, and South America, including their associated islands and regions). While it is clear that the usage of this term in the article is not the one strictly established, e.g., for legal issues, but instead is a part of a conceptual framework, I respectfully consider that the usage of the term “Americas” in the title of this article, as in other essential parts of the publication, is imprecise and contradictory.
